# Supplementary figures and images for: Effects of decision-making on indoor bouldering performances: A multi-experimental study approach
Source: PLoS One. 2021 May 13;16(5):e0250701. doi: 10.1371/journal.pone.0250701 (PMC8118292; doi:10.1371/journal.pone.0250701)

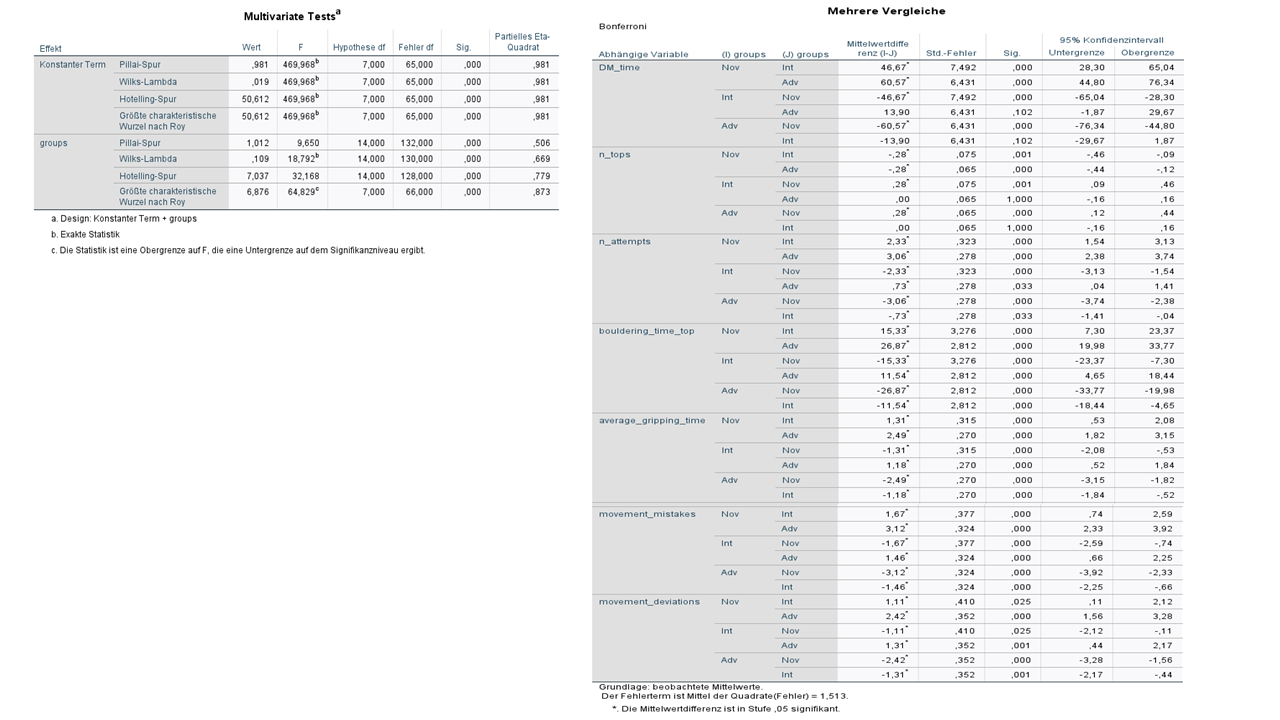

Supplement: S1 Fig — (TIF) [file pone.0250701.s001.tif]

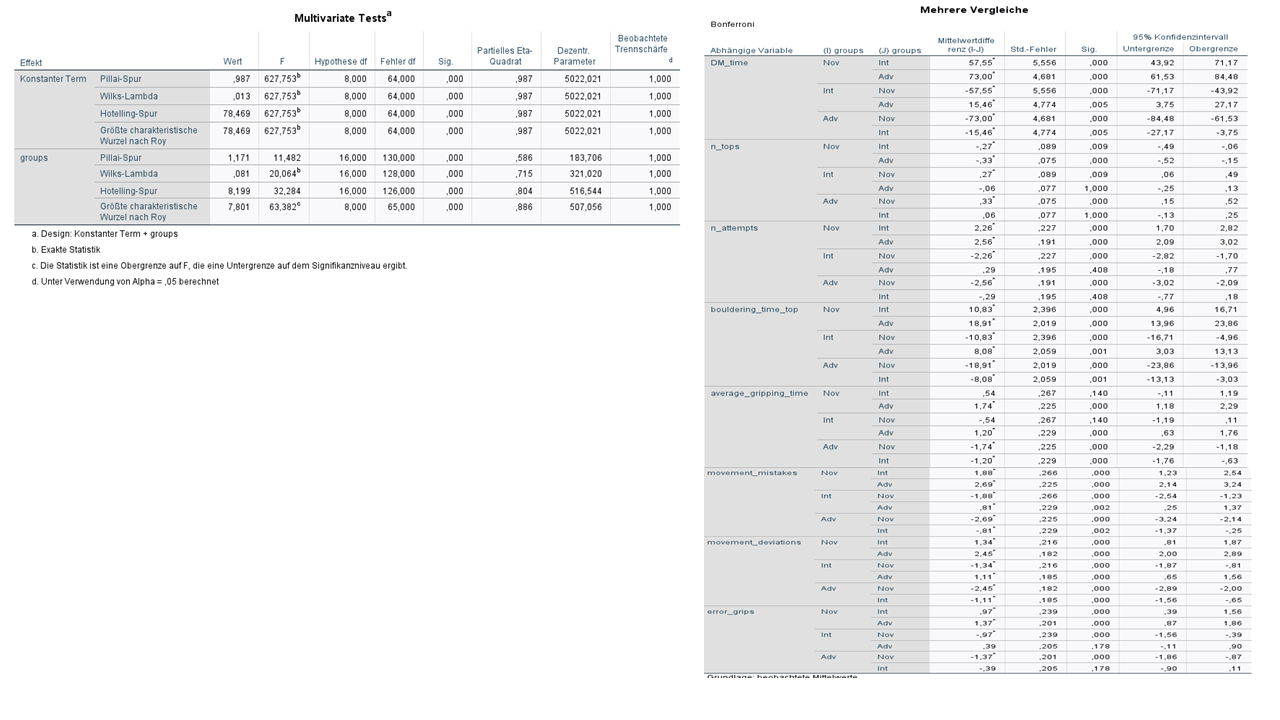

Supplement: S2 Fig — (TIF) [file pone.0250701.s002.tif]

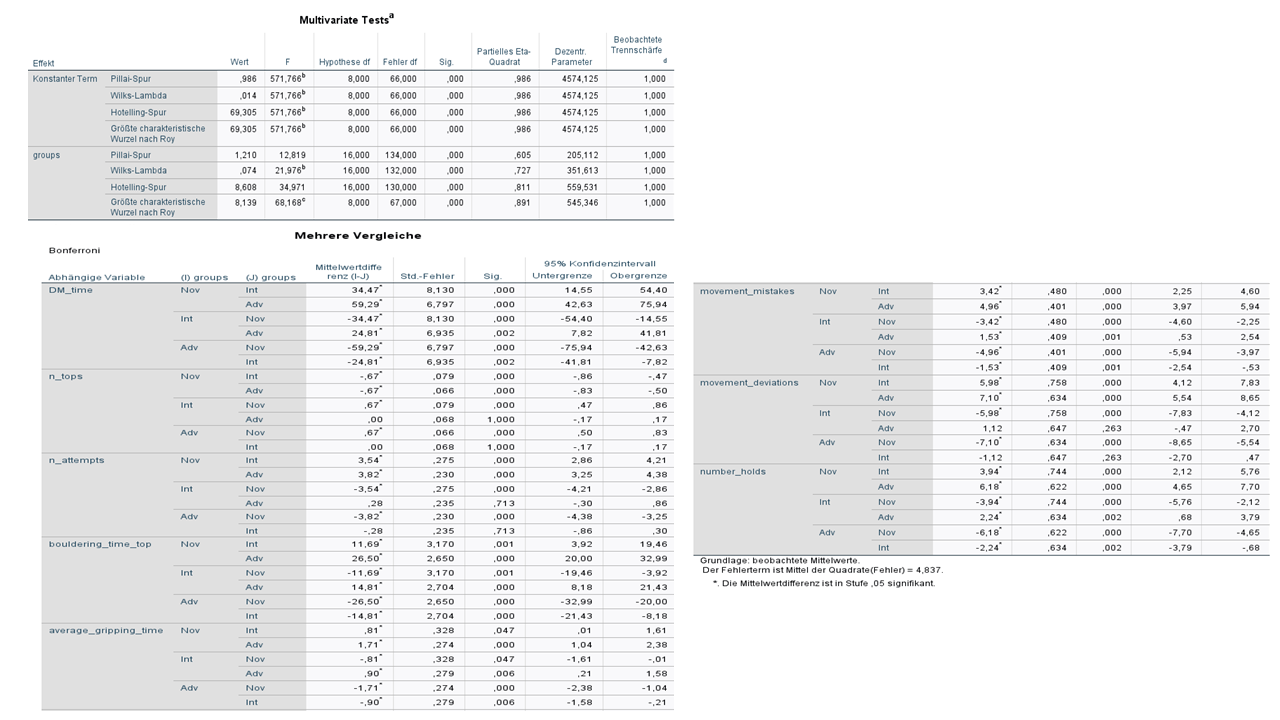

Supplement: S3 Fig — (TIF) [file pone.0250701.s003.tif]
