# Supplementary material for: Effects of decision-making on indoor bouldering performances: A multi-experimental study approach
Source: PLoS One. 2021 May 13;16(5):e0250701. doi: 10.1371/journal.pone.0250701 (PMC8118292; doi:10.1371/journal.pone.0250701)
Supplement: Attachment — Submitted filename: Response to Reviewers_2.0.docx [file pone.0250701.s004.docx]

**Response to the reviewers**

Dear Academic Editor and reviewers,

We would like to thank you for the useful comments and suggestions you made on our manuscript. Your comments were undoubtedly helpful to improve the quality of our paper.

As you hopefully will notice by reading the following response to the reviewers, we strove to implement all the recommendations made by the reviewers and to address point-to-point the reviewers’ issues.

We, therefore, hope that our revised manuscript now fully meets PLOS ONE´s publication criteria.

We are looking forward to your response.

Kind regards,

Jerry Medernach

Corresponding author

**1. Journal requirements:**

**1. Please ensure that your manuscript meets PLOS ONE's style requirements, including those for file naming.**

**Response**: We carefully re-examined the style templates and adapted the manuscript to the style requirements (see track changes).

**2. Responses to the reviewer #1**

**R1C1: I was concerned with the number of statistical tests employed in this experiment, and the fact that the same sample was used across three experiments. I think at minimum implications for Type I errors should be discussed in the limitations section. The authors might also consider adjusting their alpha level to account for multiple comparisons.**

**Response**: From a statistical point of view, the reviewer´s issue regarding the use of the same sample across the three experiments is justified and, therefore, implications for type I errors have been added to the limitations section. The number of statistical tests in the MANOVA (i.e., seven response variables) is indeed relatively high compared to the sample size in our study (also discussed in the limitations section), although a priori power analysis indicated a relatively high power of 0.8. We would like to mention that, as the cognitive processes of decision-making have not yet been investigated in indoor bouldering, our purpose was to examine all the corresponding variables to better understand the cognitive processes of decision-making in indoor bouldering, despite the higher risk of type I errors. In fact, implementing a multi-experimental design to examine the decision-making tactics of the same athletes under different experimental conditions provided useful insights (this has been added to the limitations section) in the decision-making in indoor bouldering. Furthermore, we would like to mention that recruiting participants in indoor bouldering can be challenging, particularly regarding subjects with advanced bouldering levels. Therefore, using different samples may have contributed to distinctly lower sample sizes. In this context, we think it is worthwhile to mention as well that the sample size of the present manuscript is relatively high compared to other publications in the research field. Finally, we may be wrong, but we are a bit concerned with adjusting the alpha level (i.e., 0.001) as this would raise the risk of type II errors.

**R1C2: Additionally, I was confused as to why ANOVA was chosen over linear regression to analyse the data. There are not natural divisions or groupings across skill level in climbing (and therefore the group divisions are always somewhat arbitrary), and regression would prevent arbitrary divisions in the data set. The findings in this study were not always consistent across skill level (i.e., sometimes experts outperformed both intermediates and novices, while other times experts and intermediates outperformed novices), this made the results hard to track and confusing to interpret. I think that retaining the continuous nature of the data with regression might be more informative. Further, it is harder to intuitively interpret the size of effects in ANOVA and I think using linear regression would allow for a more intuitive interpretation of the data. If ANOVA is to be used, this statistical choice should be justified, and the differential findings across groups should be further discussed.**

**Response:** As we kept the ANOVA, the differential findings across the groups were further discussed. It is true that group divisions in climbing and bouldering can be challenging and that there are no natural divisions or groupings across skill levels. Notwithstanding, we are convinced that the groupings in the present manuscript are not arbitrary and therefore justified. Table 1 displays, in addition to the bouldering ability levels, significant differences between the three ability groups for (a) the sport-specific experiences, (b) the self-perceived technical skills, and (c) the grip strength. Altogether, these parameters emphasize that there are justified differences between the three groups. Furthermore, we would like to mention that the groupings in our manuscript were based on the recommendations of the International Rock Climbing Research Association (Draper et al., 2011; Draper & Gilles, 2016) and ANOVA was chosen because working with the IRCRA´s ability groups permits a higher comparability with further studies in the research field (see l. 226). Although the regression may have circumvented the group divisions, it must, however, be considered that climbing degrees do not increase in a linear matter, which means that the differences between, for instance, a 6b Fb task, a 7b Fb task, and an 8b Fb are not linear. We, therefore, believe that a linear regression would not have consisted of the better choice because ability levels and climbing performances do not progress in a linear matter. Finally, it is true that our findings are not always consistent across skill levels (discussed in the limitations section), but we think that this is mostly related to the complexity of the tasks than the group divisions, which has been further discussed in the general discussion.

**R1C3: While I think the authors do a good job discussing decision making in sport, I think they should spend more time connecting how their measures assess decision making, as I felt this wasn’t completely clear.**

**Response:** Fair point, please refer to our adaptations in the data collection procedure section (i.e., movement analysis, l. 329).

**R1C4: I found tables in the results section useful, but overall the results section was rather cumbersome to read, and I felt interpretation of effect sizes would help break up the number of statistical tests presented, as well as help the reader understand the practical significance of the findings.**

**Response:** We agree on that point. Therefore, the effect sizes and further MANOVA results were copied to the tables (also suggested by the reviewer #3) and some results were set in brackets to make the results sections less cumbersome.

**R1C5: p. 8 line 199, affordances should be defined and Gibson should be cited. Further, I think the authors should spend more time developing how affordances relate to the theoretical concepts they are discussing and the study they are presenting. Relatedly, I would explain how affordances were made more challenging. My understanding of Gibson’s view of affordances is that we either perceive an opportunity for action or we don’t. Affordances are the interaction between the environment and the observer, they do not reside in the environment.**

**Response**: Many thanks for that point. The definition of affordance has been added and Gibson was cited. How affordances were made more challenging is explained on p. 7 (l. 185-199).

**R1C6. There were some sentences throughout that were awkwardly worded (p. 4 lines 99-108, p.6 lines 143-145 & lines 159-162).**

**Response**: Corrected, many thanks for these recommendations.

**R1C7. I might spend a bit of time justifying why bouldering is an open-skill sport. I would think it is probably somewhere on the continuum from open to closed, as many aspects of the environment are fixed in bouldering. The authors may just want to add a bit more nuance to the discussion of open/closed skill in the context of bouldering.**

**Response**: Fair point, has been further described and adapted (l. 103-110).

**R1C8. I would make sure to include effect sizes for each statistical test. For example, on p. 17 lines 438-442. These are potentially interesting results, but an effect size would aid in interpretation of these findings.**

**Response**: Done, see tables 2-4, please also refer to R1C4.

**R1C9. On page 15 line 193, why isn’t an r value included for this comparison?**

**Response**: Done. Thank you for this point.

**3. Responses to the reviewer #2**

**R2C1. The authors could note more clearly the covariates used in the statistical analysis section (e.g., did authors control for grip strength?) and report full ANOVA/MANOVA tables as an appendix).**

**Response**: Full MANOVA tables were added to the supporting information section and MANOVA results have been completed in the tables 1-4 (please also refer to R1C8). We used a MANOVA instead of a MANCOVA and thus, we did not integrate covariates into the statistical analysis. More precisely and by following Simmons et al. (2011), authors should report the statistical results of the analysis, if covariates were used, without the covariates, as reporting covariate-free results make transparent the extent to which a finding is reliant on the presence of a covariate. Therefore, the use of covariates should be justified to avoid that covariates are required for the detection of significant findings (please refer to Simmons, Nelson, and Simonsohn, 2011: False-positive psychology: undisclosed flexibility in data collection and analysis allows presenting anything as significant, Psychological Science, 22, 1359-1366).

**R2C2. The authors could consider removing statistics from their abstract, which might shorten it and make it easier to read.**

**Response**: We removed the statistics from the abstract, which made it shorter and easier to read.

**R2C3. The authors could give a very brief overview of three experiments, how they differ, and their goals in the final paragraph of the introduction.**

**Response**: Good point, we did that (l. 185-199)

**R2C4. The authors could very briefly explain the grading system for those not familiar with it.**

**Response**: Done l. 223-225.

**R2C5. The authors could explain the p-values in the tables both in the text and in the legend to each table.**

**Response**: Done, see tables 1-4.

**R2C6. The authors could confirm data availability as per this journal’s policy**

**Response**: Thanks for that point. Data availability has been confirmed in the data availability section. Further files that are not mentioned in the manuscript are available from protocols.io.

**R2C7. The authors could clarify the role of physical strength – apart from grip strength – in the sport (e.g., pull up strength) and whether this could play a role at a novice/intermediate level (if so, the authors could note if this was assessed)**

**Response**: Done l. 931-933.

**4. Responses to the reviewer #3**

**R3C1. Line 173: It would be interesting to read a clear statement of contribution before the study purpose.**

**Response:** Authors contributions are listed as per the journal´s policy in the author contribution section by using the CrediT taxonmy. The contributions were: Jerry Medernach: Conceptualization, Methodology, Investigation, Data Curation, Writing - Original Draft; Daniel Memmert: Conceptualization, Methodology, Data Curation, Writing: Review & Editing, Validation, Supervision. However, should this information really be integrated into the introduction section?

**R3C2. Line 211: Explain how the study size was arrived at. Possibly, information about sample size calculation can be moved from statistics to this section. Just a suggestion.**

**Response**: Indicated in l. 207-210 and discussed in the limitations section.

**R3C3. Lines 211-213: Describe the setting, locations, and relevant dates, including periods of recruitment, exposure, follow-up, and data collection**

**Response**: Done l. 209 and 255.

**R3C4. Lines 213-215: provide the sources and methods of selection of participants”**

**Response**: Done l. 208

**R3C5. Line 287: provide a structure in the section would help to read. Possibly, organizing by “anthropometrics and strength” and “Movement analysis” or similar”**

**Response**: Done.

**R3C6. Lines 313-326: who made the analysis? Specify the characteristics of the observer. Additionally, how accuracy and precision of data collected was ensured? Please provide information about validity and reliability of the approach.

Response**: Good point, added (l. 354-360).

**R3C7. Effect size is missing in the tables. Any possibility to include?**

**Response**: Done, please refer to the tables 2-4.

**R3C8. Text in results is sometimes repeating the information in the tables. Not necessary and can be considered to simplify.**

**Response**: Many thanks for this point. The results sections were shortened (please also refer to R1C4).

**R3C9. At the end of each discussion, a section of summary of findings, particular practical applications (for each study), and future research directions should be added, as well as identification of study limitations.**

**Response**: This is a fair point. Many thanks. Done for each discussion.
