# Supplementary material for: Effects of decision-making on indoor bouldering performances: A multi-experimental study approach
Source: PLoS One. 2021 May 13;16(5):e0250701. doi: 10.1371/journal.pone.0250701 (PMC8118292; doi:10.1371/journal.pone.0250701)
Supplement: Attachment — Submitted filename: Response to Reviewers_3.0.docx [file pone.0250701.s005.docx]

**Response to Academic Editor and Reviewer #1**

Dear reviewer #1,

After having profoundly re-read the manuscript, we agree on your comments and we would like to thank you for the useful suggestions you made. Your comments were helpful to improve the quality of our manuscript. Please find hereinafter our responses to each of your concerns. We hope that our revised manuscript now fully meets PLOS ONE´s publication criteria.

We are looking forward to your response.

Kind regards,

Dr. Jerry Medernach

Corresponding author

**Major concern #1:** “*The authors seem to be separating decision making and affordances, and I am wondering how different these two concepts are. While I am very familiar with the literature on affordances, I know less about the decision-making in sports literature. However, from the examples that the authors provide, they seem fairly similar. For example, the findings that more experienced surfers are better at determining whether or not a wave is surfable sounds almost identical to the findings in the literature on affordance perception in experts (e.g. Correia, 2012 or Weast, Shockley, & Riley, 2011). I think it would be helpful for the authors to clarify how decision making and affordances are similar or different (i.e. are there meaningful distinctions between these two terms or are two fields of researchers calling the same things by two different names) […] I think a clearer distinction between decision making and affordances will help with this. One way to frame things would be to say that perception of affordances is an initial process, and then based on the actions that a climber thinks they can complete they have to then craft those potential actions into a motor plan.*”

**Response:** We provided a more conscious description of how decision-making and affordances are related to each other and to clarify the distinction between both terms, p. 6 (l. 137-141). In addition, we removed, when inappropriate, the term of affordance across the manuscript, for instance p. 20, l. 493.

**Major concern #2:** “*In addition, throughout the authors make the argument that affordances were made “more demanding” (Page 7-8 lines 192-193 and elsewhere) and I think this is a bit of a tricky argument to make. In the view of Gibson, affordances are either perceived or they aren’t (i.e. an actor either perceives a possibility for action or they don’t), so affordance perception can’t really be made more demanding. For example, someone with a narrow body frame may perceive an aperture as passable, whereas someone with a wider body frame would not. It is not as if the affordance is more demanding for the person with a wider body frame, it just isn’t perceived. In an athletic context, a more advanced climber may perceive a very small hand hold as graspable, whereas a less experienced climber would not. The affordance is not more demanding for the less experienced climber, they just don’t see that hold as graspable. I think the modifications in experiment 2 added more opportunity for misperception of affordances, but did not make affordance perception more demanding.*”

**Response**: Many thanks for this comment. We´ve changed this across the manuscript, for instance p. 7-8, l. 192-193.

**Major concern #3:** “*I appreciated the extra explanation in the methods and results, but I am still a little stuck on the idea of a “best-solution”. There are so many variables that could impact what is someone’s own “best solution”, and I still think the use of a general “best solution” is somewhat problematic. If I am climbing, my performance does not depends on how I climb the route, but instead if I am able to make it to the top and how quickly I do so.*”

**Response**: This is a fair point and the use of a general best solution to a task can indeed be quite problematic. We more profoundly discussed the problematic of a general best solution in the discussion of Experiment 3 (p. 33, l. 797-825) and in the final limitations section (p. 38, l. 937-951). In addition, we made some changes across the manuscript to indicate more consciously that the best solution consisted of how experts perceived to best climb the tasks (e.g., p. 13, l. 320-322; p. 16, l. 409-410).

**Major concern #4:** “*I still found the results section somewhat cumbersome to read. I think a clear one sentence description of the take away from each experiment immediately following the results section would be useful. I think given the mixed findings across groups; the results sections can be hard to follow. Thus, the authors may have to put in some extra effort to make their findings clear and easy to follow.*”

**Response**: Thank you for this suggestion. We adapted the result sections across the manuscript (shorter and less cumbersome to read) and restructured the discussion sections with a clear statement of the takeaway message in each experiment. We believe that the results are now more consciously and less cumbersome to read.

**Minor concern #1: “***Page 3 line 74, what does “more appropriate” mean?*

**Response**: Changed, p. 3, l. 71-75.

**Minor concern #2:** “*Page 6 line 140 “could observe” should be “observed”, same on line 147 and 151, as well as in a few other places throughout the paper*”

**Response**: We´ve changed that across the manuscript.
